# The thermal dependency of locomotor performance evolves rapidly within an invasive species

**DOI:** 10.1002/ece3.3996

**Published:** 2018-04-02

**Authors:** Georgia K. Kosmala, Gregory P. Brown, Keith A. Christian, Cameron M. Hudson, Richard Shine

**Affiliations:** ^1^ School of Life and Environmental Sciences The University of Sydney Sydney NSW Australia; ^2^ Research Institute for the Environment and Livelihoods Charles Darwin University Darwin NT Australia

**Keywords:** abiotic challenges, adaptation, common‐garden, locomotor performance, *Rhinella marina*

## Abstract

Biological invasions can stimulate rapid shifts in organismal performance, via both plasticity and adaptation. We can distinguish between these two proximate mechanisms by rearing offspring from populations under identical conditions and measuring their locomotor abilities in standardized trials. We collected adult cane toads (*Rhinella marina*) from invasive populations that inhabit regions of Australia with different climatic conditions. We bred those toads and raised their offspring under common‐garden conditions before testing their locomotor performance. At high (but not low) temperatures, offspring of individuals from a hotter location (northwestern Australia) outperformed offspring of conspecifics from a cooler location (northeastern Australia). This disparity indicates that, within less than 100 years, thermal performance in cane toads has adapted to the novel abiotic challenges that cane toads have encountered during their invasion of tropical Australia.

## 1 INTRODUCTION

Environmental conditions can impose strong selective pressure on the biology of organisms, shaping the range of ambient conditions over which an animal can function (Angilletta, Niewiarowski, & Navas, 2002; Angilletta, Steury, & Sears, 2004; Huey & Kingsolver, 1989). An individual's performance (such as its locomotor ability) typically depends upon its physiological state, and factors such as body temperature can modify an animal's ability to perform ecologically important functions such as sprinting, climbing, or burrowing (e.g., Kaufmann & Bennett, 1989; Londos & Brooks, 1988; Seebacher & Franklin, 2011; Tingley, Greenlees, & Shine, 2012). We thus expect natural selection to fine‐tune those responses such that animals are capable of effective performance under the conditions that they encounter within their natural habitat (e.g., Eliason et al., 2011). Consistent with that hypothesis, comparisons among species, and among geographically separate populations within wide‐ranging species, often report divergences in thermal performance curves among populations that inhabit areas with different thermal conditions (Beuchat, Pough, & Stewart, 1984; Eliason et al., 2011; Feder, 1978; Gilbert, Huey, & Gilchrist, 2001; Llewelyn, Phillips, Alford, Schwarzkopf, & Shine, 2010; Titon, Navas, Jim, & Gomes, 2010; Wilson, 2001). Individuals from a hotter area outperform conspecifics from a colder area (in speed, endurance, etc.) when tested at a high temperature, whereas the reverse is true if the test is conducted at a low temperature (Beuchat et al., 1984; Gilbert et al., 2001; McCann, Greenlees, Newell, & Shine, 2014; Titon et al., 2010; Wilson, 2001; Winwood‐Smith, Alton, Franklin, & White, 2015).

Adjusting performance optima to local conditions may be achieved via thermal plasticity (within‐generation phenotypic plasticity, whereby environmental conditions directly induce shifts in thermal performance curves) or adaptation (across‐generation shifts in the relative frequencies of alleles that optimize performance under the conditions most often encountered) (Beuchat et al., 1984; Gilbert et al., 2001; Maron, Elmendorf, & Vilà, 2007; Maron, Vila, Bommarco, Elmendorf, & Beardsley, 2004; McCann et al., 2014; Seiter & Kingsolver, 2013; Titon et al., 2010; Wilson, 2001; Winwood‐Smith et al., 2015). Studies of wild‐caught individuals can demonstrate phenotypically plastic responses, but cannot unequivocally test the role of adaptive change in generating geographic divergence in thermal performance curves (e.g., Kosmala, Christian, Brown, & Shine, 2017). To demonstrate that geographic divergence in performance optima is due to across‐generation shifts, we need to measure the performance of offspring from different populations that have been reared under standard (“common‐garden”) conditions (Hoffmann & Merilä, 1999; Hudson, Brown, & Shine, 2017; Phillips, Brown, Webb, & Shine, 2006).

Many cases of intraspecific variation in thermal performance curves likely involve adaptation as well as thermal plasticity (Gilbert et al., 2001), but the issue is especially interesting in the case of populations that have diverged only recently from a common ancestor (Eliason et al., 2011; Gilbert et al., 2001). In such a case, we can explore the potential for populations to adapt rapidly to novel challenges (Huey & Kingsolver, 1989; Maron et al., 2004, 2007; McCann et al., 2014; Seiter & Kingsolver, 2013; Tingley et al., 2012; Winwood‐Smith et al., 2015). Such an ability might be critical to population viability if conditions change, as may be the case for many species under anthropogenically induced climate change. Invasive species from plants to vertebrates offer robust model systems to examine the rate of adaptive change as they colonize new regions (Brown, Phillips, Dubey, & Shine, 2015; Llewellyn, Thompson, Brown, Phillips, & Shine, 2012; Maron et al., 2004, 2007; Phillips et al., 2006; Tingley & Shine, 2011; Tingley, Vallinoto, Sequeira, & Kearney, 2014; Tingley et al., 2012). In many cases, such taxa exhibit rapid shifts in phenotypic traits due to adaptation rather than (or as well as) plasticity (Brown et al., 2015; Llewellyn et al., 2012; Maron et al., 2004, 2007; McCann et al., 2014; Seiter & Kingsolver, 2013; Tingley et al., 2012).

The cane toad (*Rhinella marina*, formerly *Bufo marinus*) is a large bufonid anuran native to Latin America, but intentionally translocated to Australia in 1935 in a misguided attempt to control insect pests of commercial agriculture (Lever, 2001). The toads have since spread widely across Australia (Urban, Phillips, Skelly, & Shine, 2007), colonizing new environments. In the process, toads have undergone substantial shifts in morphology (Hudson, Brown, & Shine, 2016; Hudson, McCurry, Lundgren, McHenry, & Shine, 2016; Phillips et al., 2006), physiology (Brown & Shine, 2014; Brown et al., 2015; Llewellyn et al., 2012), and behavior (Brown et al., 2015; Gruber, Brown, Whiting, & Shine, 2017; Hudson et al., 2017). Studies on captive‐reared progeny of toads collected in different parts of Australia have confirmed that many of those divergences are heritable (Gruber et al., 2017; Hudson, Brown, et al., 2016; Hudson et al., 2017) and, hence, are likely to be the result of adaptation. In recent work, we documented significant differences in thermal and hydric performance curves between wild‐caught cane toads from different parts of Australia, as well as between Australian populations and those from the native range in Brazil, and a stepping‐stone population in Hawai'i (Kosmala et al., 2017). To clarify the degree to which such divergences are a result of adaptation rather than plasticity, we raised toads from eastern and Western Australia under standard (“common‐garden”) conditions and measured their locomotor abilities under a range of thermal and hydric conditions.

## 2 METHODS

### 2.1 Study species and collection localities

Cane toads (*Rhinella marina*) are large “true toads” of the family Bufonidae (Lever, 2001). The species’ native range encompasses parts of Mexico, southern Texas, and Central and South America (Easteal, 1981; Lever, 2001; Zug & Zug, 1979). Commercial sugarcane growers imported toads from French Guiana to control insect pests in plantations in Puerto Rico in the 1920s (Lever, 2001). From there, 150 toads were translocated to the island of Honolulu, Hawai'i, in 1932 and released in sugarcane fields (Easteal, 1981; Lever, 2001; Zug & Zug, 1979). Three years later, 101 descendants of the Hawai'ian immigrants were collected and shipped to northeastern Australia, where their progeny were released along the Queensland coast (Easteal, 1981).

**Figure 1:**
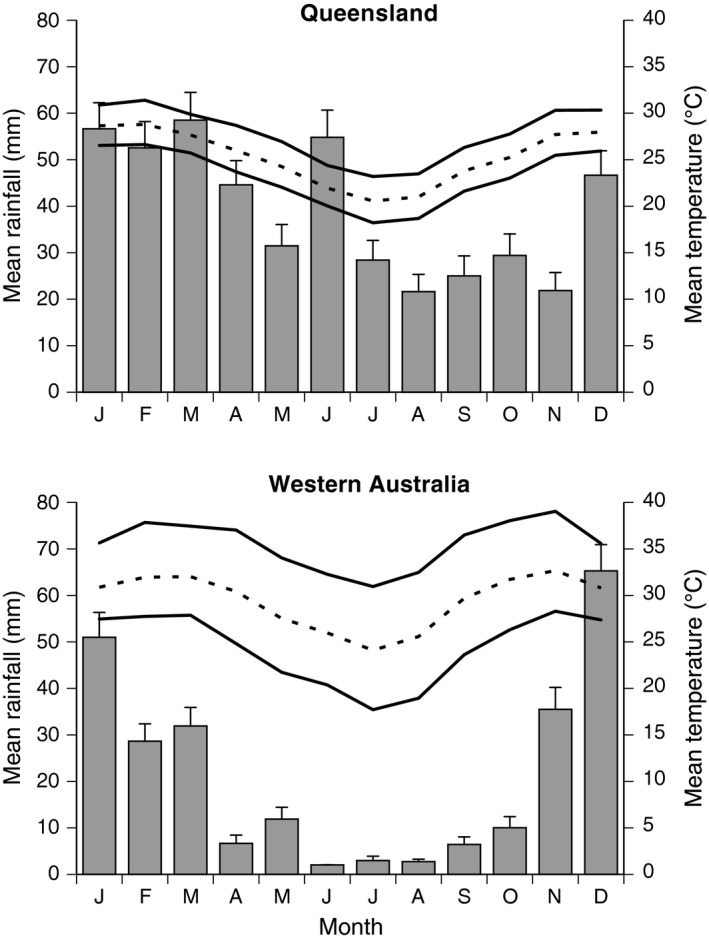
Average climatic conditions in sites where adult cane toads were collected (i.e., the parents of the progeny studied in the present paper). The graphs show monthly average (dashed line), maximum and minimum (solid lines) of temperature and precipitation (bars with standard errors) based on data for three sites in Queensland (Townsville, Innisfail, and Tully) and four sites in Western Australia (El Questro, Purnululu, Wyndham, and Oombulgurri)

In the course of their Australian invasion, toads have spread from thermally mild, high‐precipitation regions of Queensland into hotter and seasonally arid regions of Western Australia (Kearney et al., 2008; Shine, 2010). We sampled cane toads from both of these climatic areas. Our three Queensland sites (QLD), located close to where the species was originally introduced (Townsville [GPS −19.257627, 146.817871], Innisfail [GPS −17.524681, 146.032329], Tully [GPS −17.932869, 145.923556]), experience moist and relatively mild conditions year‐round, whereas the four collection sites in Western Australia (WA; El Questro [GPS −16.008438, 127.979811], Purnululu [GPS −17.529752, 128.400838], Wyndham [GPS −15.464803, 128.100143], Oombulgurri [GPS −15.180417, 127.845039]) were colonized by toads only recently (<10 years ago: Kearney et al., 2008) and experience hotter and seasonally arid conditions (Figure 1). Monthly average temperatures are approximately 3°C hotter in our WA sites than in our QLD sites, and the WA sites average <20 mm of rain in 7 months of the year whereas all months fall above this level at our QLD sites (Figure 1). In summary, our QLD sites provide a moister, cooler, and less seasonal environment than our WA sites.

### 2.2 Methods of obtaining specimens for study

Toads were collected by hand at night, placed in damp cloth bags, kept cool to reduce stress, and transported to local laboratory facilities. They were allocated to 1‐m^2^ enclosures fitted with sprinklers and lights for ad libitum water and naturally occurring prey, until mating. As the enclosures were located outdoors at a field station in the Northern Territory (12°37′S 131°18′E; an environment characterized by warm temperatures year‐round, but clearly defined wet and dry seasons), toads were exposed to natural thermal conditions. We injected pairs of toads from the same populations of origin with 1 ml of leuprorelin acetate (Lucrin, Abbott Australasia, Botany, NSW; 1:20 dilution, 0.7 ml for females and 0.3 ml for males) to induce spawning (for detailed methods, see Phillips et al., 2010). Eggs were moved to 20‐L outdoor bins, fitted with water pumps, netted lids to exclude predators, and containing local aquatic plants as a food source. These bins were checked daily for metamorphic toads, which were then moved to more appropriate cages. Metamorphic young were raised in captivity in outdoor enclosures at the same field station, in groups of 30 per 7‐L container with soil and ad libitum water, and were fed termites daily. Groups were split into smaller units as toads grew, to avoid competition for food and cannibalism, and eventually moved to enclosures with the same setup as the parental ones until the young toads attained sexual maturity (at 18–20 months of age, 96.3 ± 1.2 mm mean snout‐vent length [SVL] ± *SE*). The common‐garden toads were kept in the same bins as described above, under ambient temperature conditions, from the time of breeding of their parents (mid 2014) until trials for this study were conducted (November 2016); during this period, average minimum monthly temperature was 20.1°C (±0.74 *SE*) and average monthly maximum temperature was 35.1°C (±0.31 *SE*). There were no significant differences in average minimum or maximum rearing temperatures between toads originating from WA versus QLD parents (both *F*_1,17_ < 0.38, both *p > *.55).

We tested 32 progeny from these matings (8 males and 8 females from Queensland and the same number from Western Australia), from a total of 19 captivity‐laid clutches (4 from Townsville, 4 from Innisfail, 3 from Tully, 2 from El Questro, 2 from Purnululu, 2 from Wyndham, and 2 from Oombulgurri). For detailed methods, see Hudson, Brown, et al. (2016).

### 2.3 Methods of experimental testing

For 1 week prior to testing (and between trials), we kept toads in the laboratory, fed them with crickets, and provided ad libitum access to water and shelter. The room was maintained at 25°C, with a 12:12‐hr light cycle. Prior to trials, we kept the toads in water‐filled containers at the test temperature for a minimum of 2 hr. After emptying the toads’ bladders by manual pressure on the abdomen, we encouraged the animals to run along a circular wooden track inside the temperature‐controlled room for 10 min, stimulating them to keep moving by administering gentle pokes to their urostyles. A trial concluded after 10 min of stimulation, or when the animal refused to move even after 1 min of stimulation. We recorded total distances moved in terms of body lengths travelled during the trial period. After these initial trials, we placed toads in desiccating conditions (exposed to a flow of dry air) and allowed them to dehydrate overnight until they lost 10% of their initial body mass. We then repeated the locomotor test protocol over the following days at 90% and 80% of the initial body mass. This protocol was performed at 15, 25, and 35°C, with a week of rest and rehydration in between trials at different temperatures. This protocol is described in more detail by Kosmala et al. (2017). All procedures were approved by the University of Sydney Animal Care and Ethics Committee (Protocol # 703) and by Charles Darwin University Animal Ethics and Welfare Committee (AEWC) (Protocol # A13016).

### 2.4 Statistical analyses

These procedures generated data on the influence of the three categorical variables (test temperature, hydration level, and population of parental origin) on locomotor performance of toads raised in captivity. Data for all analyses conformed to assumptions of normality and homoscedastic variances.

We used linear mixed models (package lme4) in the open‐access software R Studio version 0.99.893 (R Core Development Team, 2013), to evaluate the fixed effects of test conditions (temperature and hydration level) and population of parental origin on the toads’ locomotor performance. We included individual toad ID as a random factor in the analyses to accommodate multiple measures taken from individual toads. We also included clutch nested within population as a random effect to model relatedness among siblings. We treated test temperature and hydration level as three‐level categorical variables.

## 3 RESULTS

Mean body size (SVL) did not differ significantly between toads with parents originating from QLD versus WA (98.1 vs. 98.4 mm, respectively, *F*_1,29_ = 0.017, *p *=* *.90). Further, our measure of locomotor performance (body lengths/min) was independent of SVL (*F*_1,28_ = 1.42, *p *=* *.24). Although mean parental SVL exhibited a significant interaction between state of origin and sex (*F*_1,1_ = 7.54, *p *=* *.009), progeny SVL was not significantly affected by parental SVL (interaction, *F*_2,2_ = 0.99, *p *=* *.39).

**Figure 2:**
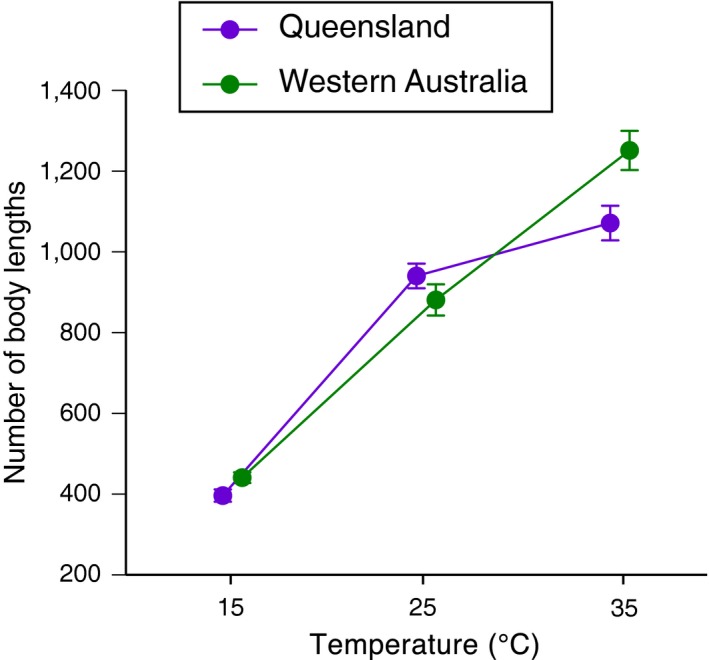
Effect of test temperature on locomotor performance of cane toads that were raised in captivity, but whose parents were collected from two different regions within Australia (Queensland and Western Australia). The *Y*‐axis shows the total distance moved during a 10‐min trial, expressed as body lengths. Graph shows mean values and standard errors

Temperature interacted with population (*F*_2,205_ = 10.25, *p *<* *.0001; Table S1) in its effect on locomotor performance. Toads from both populations performed equally well at temperatures of 15°C and 25°C, but at the highest test temperature (35°C), offspring of WA parents travelled significantly farther than did the offspring of QLD parents (Figure 2). Hydration level had no significant effect on locomotor performance. The three‐way interaction (temperature*hydration*population) was nonsignificant (*F*_4,200_ = 0.0981, *p *=* *.9830) and, thus, removed from the analysis (Table S1).

## 4 DISCUSSION

In a previous study, we showed that locomotor performance of wild‐caught cane toads differed among populations from Brazil, Hawai'i, and four locations within Australia (Kosmala et al., 2017). However, we were unable to identify the proximate basis for that divergence; it might have been entirely due to toads acclimating to the conditions they experience during ontogeny. The present study extends the earlier work by showing that the divergence between two of the Australian locations (WA vs. QLD) is underpinned at least partly by adaptation. Even when raised under standard conditions, cane toads whose parents were collected in different parts of Australia differed in the degree to which their locomotor ability was affected by temperature. When tested under hot conditions, toads whose parents were collected from a hot‐climate location exhibited better locomotor performance than did toads whose parents were collected from a cooler area. However, locomotor performance of toads from the two populations was near identical at lower test temperatures (Figure 2). This is exactly the pattern that one would predict if toads adapt to the thermal conditions encountered within their local area.

Introduced species exhibit both rapid evolution and phenotypic plasticity to respond to the novel conditions they encounter in the introduced range (Maron et al., 2004, 2007; Seiter & Kingsolver, 2013). An adaptive basis to geographic divergence in thermal performance curves, such that local populations function best under the conditions they are most likely to experience, is consistent with an extensive literature (see Introduction). However, the timescale of that adaptive process is much shorter in the current study than in prior comparisons. Cane toads had been present in Australia for only 80 years at the time we collected the parental stock and had been encountering the hot dry conditions of northwestern Australia for only 8 years at the invasion front (Hudson, Brown, et al., 2016; Kearney et al., 2008). Despite their low genetic diversity (due to founder effects in successive translocations: Slade & Moritz, 1998; Rollins, Richardson, & Shine, 2015), cane toads in Australia have evolved in ways that enhance their ability to thrive in their new range. As the invasion expanded westwards, it moved into habitats that were more extreme (both thermally and hydrically) than occur within the species’ native range (Tingley & Shine, 2011; Tingley et al., 2012, 2014); and in response to those novel challenges, cane toads have evolved to function more effectively at higher temperatures than they were capable of when they first colonized Australia (as inferred from toad performance in long‐colonized regions). In combination with evidence of local adjustment of the critical thermal minimum to low temperatures in southern Australia (McCann et al., 2014) and of water balance to arid conditions in central Queensland (Tingley et al., 2012), our study suggests that despite the brief time span involved, cane toads are rapidly changing in ways that enable them to better exploit the novel challenges and opportunities within the Australian continent.

## DATA ACCESSIBILITY

Data will be available at the external repository Dryad.

## CONFLICT OF INTEREST

None declared.

## AUTHOR CONTRIBUTIONS

GKK carried out all animal experimentation, participated in data analysis and design of the study, and drafted the manuscript; GPB carried out statistical analyses and interpretation of the data; CMH carried out fieldwork and breeding; KC provided logistical support and equipment for experimentation and helped draft and revise the article; RS conceived the study, participated in the design of the study, coordinated the study, and helped draft the manuscript. All authors gave final approval for publication.

## References

[ece33996-bib-0001] Angilletta, M. J. , Niewiarowski, P. H. , & Navas, C. A. (2002). The evolution of thermal physiology in ectotherms. Journal of Thermal Biology, 27, 249–268. https://doi.org/10.1016/S0306-4565(01)00094-8

[ece33996-bib-0002] Angilletta, M. J. Jr , Steury, T. D. , & Sears, M. W. (2004). Temperature, growth rate, and body size in ectotherms: Fitting pieces of a life‐history puzzle. Integrative and Comparative Biology, 44, 498–509. https://doi.org/10.1093/icb/44.6.498 21676736 10.1093/icb/44.6.498

[ece33996-bib-0003] Beuchat, C. A. , Pough, F. H. , & Stewart, M. M. (1984). Response to simultaneous dehydration and thermal stress in three species of Puerto Rican frogs. Journal of Comparative Physiology B, 154, 579–585. https://doi.org/10.1007/BF00684411

[ece33996-bib-0004] Brown, G. P. , Phillips, B. L. , Dubey, S. , & Shine, R. (2015). Invader immunology: Invasion history alters immune system function in cane toads (*Rhinella marina*) in tropical Australia. Ecology Letters, 18, 57–65. https://doi.org/10.1111/ele.12390 25399668 10.1111/ele.12390

[ece33996-bib-0005] Brown, G. P. , & Shine, R. (2014). Immune response varies with rate of dispersal in invasive cane toads (*Rhinella marina*). PLoS ONE, 9, e99734 https://doi.org/10.1371/journal.pone.0099734 24936876 10.1371/journal.pone.0099734 PMC4061023

[ece33996-bib-0006] Easteal, S. (1981). The history of introductions of *Bufo marinus* (Amphibia: Anura): A natural experiment in evolution. Biological Journal of the Linnean Society, 16, 93–113. https://doi.org/10.1111/j.1095-8312.1981.tb01645.x

[ece33996-bib-0007] Eliason, E. J. , Clark, T. D. , Hague, M. J. , Hanson, L. M. , Gallagher, Z. S. , Jeffries, K. M. , … Farrell, A. P. (2011). Differences in thermal tolerance among sockeye salmon populations. Science, 332, 109–112. https://doi.org/10.1126/science.1199158 21454790 10.1126/science.1199158

[ece33996-bib-0008] Feder, M. E. (1978). Environmental variability and thermal acclimation in neotropical and temperate zone salamanders. Physiological Zoology, 51, 7–16. https://doi.org/10.1086/physzool.51.1.30158660

[ece33996-bib-0009] Gilbert, P. , Huey, R. B. , & Gilchrist, G. W. (2001). Locomotor performance of *Drosophila melanogaster*: Interactions among developmental and adult temperatures, age, and geography. Evolution, 55, 205–209. https://doi.org/10.1111/j.0014-3820.2001.tb01286.x 11263741 10.1111/j.0014-3820.2001.tb01286.x

[ece33996-bib-0010] Gruber, J. , Brown, G. , Whiting, M. J. , & Shine, R. (2017). Geographic divergence in dispersal‐related behaviour in cane toads from range‐front versus range‐core populations in Australia. Behavioral Ecology and Sociobiology, 71, 38 https://doi.org/10.1007/s00265-017-2266-8

[ece33996-bib-0011] Hoffmann, A. A. , & Merilä, J. (1999). Heritable variation and evolution under favourable and unfavourable conditions. Trends in Ecology & Evolution, 14, 96–101. https://doi.org/10.1016/S0169-5347(99)01595-5 10322508 10.1016/s0169-5347(99)01595-5

[ece33996-bib-0012] Hudson, C. M. , Brown, G. P. , & Shine, R. (2016). It is lonely at the front: Contrasting evolutionary trajectories in male and female invaders. Royal Society Open Science, 3, 160687 https://doi.org/10.1098/rsos.160687 28083108 10.1098/rsos.160687 PMC5210690

[ece33996-bib-0013] Hudson, C. M. , Brown, G. P. , & Shine, R. (2017). Evolutionary shifts in anti‐predator responses of invasive cane toads (*Rhinella marina*). Behavioral Ecology and Sociobiology, 71, 134 https://doi.org/10.1007/s00265-017-2367-4

[ece33996-bib-0014] Hudson, C. M. , McCurry, M. R. , Lundgren, P. , McHenry, C. R. , & Shine, R. (2016). Constructing an invasion machine: The rapid evolution of a dispersal‐enhancing phenotype during the cane toad invasion of Australia. PLoS ONE, 11, e0156950 https://doi.org/10.1371/journal.pone.0156950 27658247 10.1371/journal.pone.0156950 PMC5033235

[ece33996-bib-0015] Huey, R. B. , & Kingsolver, J. G. (1989). Evolution of thermal sensitivity of ectotherm performance. Trends in Ecology & Evolution, 4, 131–135. https://doi.org/10.1016/0169-5347(89)90211-5 21227334 10.1016/0169-5347(89)90211-5

[ece33996-bib-0016] Kaufmann, J. S. , & Bennett, A. F. (1989). The effect of temperature and thermal acclimation on locomotor performance in *Xantusia vigilis*, the desert night lizard. Physiological Zoology, 62, 1047–1058. https://doi.org/10.1086/physzool.62.5.30156195

[ece33996-bib-0017] Kearney, M. , Phillips, B. L. , Tracy, C. R. , Christian, K. A. , Betts, G. , & Porter, W. P. (2008). Modelling species distributions without using species distributions: The cane toad in Australia under current and future climates. Ecography, 31, 423–434. https://doi.org/10.1111/j.0906-7590.2008.05457.x

[ece33996-bib-0018] Kosmala, G. , Christian, K. , Brown, G. , & Shine, R. (2017). Locomotor performance of cane toads differs between native‐range and invasive populations. Royal Society Open Science, 4, 170517 https://doi.org/10.1098/rsos.170517 28791174 10.1098/rsos.170517 PMC5541569

[ece33996-bib-0019] Lever, C. (2001). The cane toad. The history and ecology of a successful colonist. Otley, UK: Westbury Academic and Scientific Publishing.

[ece33996-bib-0020] Llewellyn, D. , Thompson, M. B. , Brown, G. P. , Phillips, B. L. , & Shine, R. (2012). Reduced investment in immune function in invasion‐front populations of the cane toad (*Rhinella marina*) in Australia. Biological Invasions, 14, 999–1008. https://doi.org/10.1007/s10530-011-0135-3

[ece33996-bib-0021] Llewelyn, J. , Phillips, B. L. , Alford, R. A. , Schwarzkopf, L. , & Shine, R. (2010). Locomotor performance in an invasive species: Cane toads from the invasion front have greater endurance, but not speed, compared to conspecifics from a long‐colonised area. Oecologia, 162, 343–348. https://doi.org/10.1007/s00442-009-1471-1 19841946 10.1007/s00442-009-1471-1

[ece33996-bib-0022] Londos, P. L. , & Brooks, R. J. (1988). Effect of temperature acclimation on locomotory performance curves in the toad, *Bufo woodhousii woodhousii* . Copeia, 1988, 26–32. https://doi.org/10.2307/1445918

[ece33996-bib-0023] Maron, J. L. , Elmendorf, S. C. , & Vilà, M. (2007). Contrasting plant physiological adaptation to climate in the native and introduced range of *Hypericum perforatum* . Evolution, 61, 1912–1924. https://doi.org/10.1111/j.1558-5646.2007.00153.x 17683433 10.1111/j.1558-5646.2007.00153.x

[ece33996-bib-0024] Maron, J. L. , Vila, M. , Bommarco, R. , Elmendorf, S. , & Beardsley, P. (2004). Rapid evolution of an invasive plant. Ecological Monographs, 74, 261–280. https://doi.org/10.1890/03-4027

[ece33996-bib-0025] McCann, S. , Greenlees, M. J. , Newell, D. , & Shine, R. (2014). Rapid acclimation to cold allows the cane toad to invade montane areas within its Australian range. Functional Ecology, 28, 1166–1174. https://doi.org/10.1111/1365-2435.12255

[ece33996-bib-0026] Phillips, B. L. , Brown, G. P. , Webb, J. K. , & Shine, R. (2006). Invasion and the evolution of speed in toads. Nature, 439, 803 https://doi.org/10.1038/439803a 16482148 10.1038/439803a

[ece33996-bib-0027] Phillips, B. L. , Kelehear, C. , Pizzatto, L. , Brown, G. P. , Barton, D. , & Shine, R. (2010). Parasites and pathogens lag behind their host during periods of host range advance. Ecology, 91, 872–881. https://doi.org/10.1890/09-0530.1 20426344 10.1890/09-0530.1

[ece33996-bib-0028] R Core Development Team (2013). R: A language and environment for statistical computing. Vienna, Austria: R Foundation for Statistical Computing Retrieved from http://www.R-project.org/. Accessed 2 April, 2016.

[ece33996-bib-0029] Rollins, L. A. , Richardson, M. F. , & Shine, R. (2015). A genetic perspective on rapid evolution in cane toads (*Rhinella marina*). Molecular Ecology, 24, 2264–2276. https://doi.org/10.1111/mec.13184 25894012 10.1111/mec.13184

[ece33996-bib-0030] Seebacher, F. , & Franklin, C. E. (2011). Physiology of invasion: Cane toads are constrained by thermal effects on physiological mechanisms that support locomotor performance. Journal of Experimental Biology, 214, 1437–1444. https://doi.org/10.1242/jeb.053124 21490252 10.1242/jeb.053124

[ece33996-bib-0031] Seiter, S. , & Kingsolver, J. (2013). Environmental determinants of population divergence in life‐history traits for an invasive species: Climate, seasonality and natural enemies. Journal of Evolutionary Biology, 26, 1634–1645. https://doi.org/10.1111/jeb.12159 23859223 10.1111/jeb.12159

[ece33996-bib-0032] Shine, R. (2010). The ecological impact of invasive cane toads (*Bufo marinus*) in Australia. Quarterly Review of Biology, 85, 253–291. https://doi.org/10.1086/655116 20919631 10.1086/655116

[ece33996-bib-0033] Slade, R. W. , & Moritz, C. (1998). Phylogeography of *Bufo marinus* from its natural and introduced ranges. Proceedings of the Royal Society B, 265, 769–777. https://doi.org/10.1098/rspb.1998.0359 9628036 10.1098/rspb.1998.0359 PMC1689048

[ece33996-bib-0034] Tingley, R. , Greenlees, M. J. , & Shine, R. (2012). Hydric balance and locomotor performance of an anuran (*Rhinella marina*) invading the Australian arid zone. Oikos, 121, 1959–1965. https://doi.org/10.1111/j.1600-0706.2012.20422.x

[ece33996-bib-0035] Tingley, R. , & Shine, R. (2011). Desiccation risk drives the spatial ecology of an invasive anuran (*Rhinella marina*) in the Australian semi‐desert. PLoS ONE, 6, e25979 https://doi.org/10.1371/journal.pone.0025979 22043300 10.1371/journal.pone.0025979 PMC3197141

[ece33996-bib-0036] Tingley, R. , Vallinoto, M. , Sequeira, F. , & Kearney, M. R. (2014). Realized niche shift during a global biological invasion. Proceedings of the National Academy of Sciences of the United States of America, 111, 10233–10238. https://doi.org/10.1073/pnas.1405766111 24982155 10.1073/pnas.1405766111 PMC4104887

[ece33996-bib-0037] Titon, B. Jr , Navas, C. A. , Jim, J. , & Gomes, F. R. (2010). Water balance and locomotor performance in three species of neotropical toads that differ in geographical distribution. Comparative Biochemistry and Physiology A, 156, 129–135. https://doi.org/10.1016/j.cbpa.2010.01.009 10.1016/j.cbpa.2010.01.009 20096361

[ece33996-bib-0038] Urban, M. C. , Phillips, B. L. , Skelly, D. K. , & Shine, R. (2007). The cane toad's (*Chaunus [Bufo] marinus*) increasing ability to invade Australia is revealed by a dynamically updated range model. Proceedings of the Royal Society B, 274, 1413–1419. https://doi.org/10.1098/rspb.2007.0114 17389221 10.1098/rspb.2007.0114 PMC2176198

[ece33996-bib-0039] Wilson, R. S. (2001). Geographic variation in thermal sensitivity of jumping performance in the frog *Limnodynastes peronii* . Journal of Experimental Biology, 204, 4227–4236. 11815647 10.1242/jeb.204.24.4227

[ece33996-bib-0040] Winwood‐Smith, H. S. , Alton, L. A. , Franklin, C. E. , & White, C. R. (2015). Does greater thermal plasticity facilitate range expansion of an invasive terrestrial anuran into higher latitudes? Conservation Physiology, 3, cov10 https://doi.org/10.1093/conphys/cov10 10.1093/conphys/cov010 PMC4778455 27293695

[ece33996-bib-0041] Zug, G. R. , & Zug, P. B. (1979). The marine toad, *Bufo marinus*: A natural history resumé of native populations. Smithsonian Contributions to Zoology, 284, 1–58.

