# Supplementary material for: The thermal dependency of locomotor performance evolves rapidly within an invasive species
Source: Ecol Evol. 2018 Apr 2;8(9):4403–8. doi: 10.1002/ece3.3996 (PMC5938468; doi:10.1002/ece3.3996)
Supplement: Supplementary file 1 [file ECE3-8-4403-s001.docx]

**SUPPORTING INFORMATION**

**TABLE S1** Results of statistical model on the locomotor performance (distance traveled, in terms of body lengths) of “common-garden” cane toads (raised in captivity) as a function of test conditions (levels of temperature and hydration), and the toads’ populations of parental origin. Toad ID and Clutch were included as random effects in the model and comprised 35.8% and 19.7% of the overall variance, respectively. Bold font indicates significant differences (*p* < 0.05).

| *Source* | *Degrees of freedom (df)* | *F*-value | *p-value* |
| --- | --- | --- | --- |
| Population | 1,24 | 0.6844 | 0.4163 |
| Temperature | 2,203 | 155.9268 | **<0.0001** |
| Hydration | 2,202 | 0.3584 | 0.6993 |
| Temperature*Hydration | 4,203 | 1.7177 | 0.1474 |
| Population*Temperature | 2,205 | 10.2539 | **<0.0001** |
| Population*Hydration | 2,203 | 0.6531 | 0.5215 |
